# Integrative analyses of genetic variation, epigenetic regulation, and the transcriptome to elucidate the biology of platinum sensitivity

**DOI:** 10.1186/1471-2164-15-292

**Published:** 2014-04-16

**Authors:** Bonnie LaCroix, Eric R Gamazon, Divya Lenkala, Hae Kyung Im, Paul Geeleher, Dana Ziliak, Nancy J Cox, Rong Stephanie Huang

**Affiliations:** 1 Section of Hematology/Oncology, Department of Medicine, University of Chicago, 900 E 57th street, KCBD room 7148, Chicago, IL, 60637, USA; 2 Section of Genetic Medicine, Department of Medicine, Chicago, IL, 60637, USA; 3 Department of Health Studies, The University of Chicago, Chicago, IL, 60637, USA

**Keywords:** microRNA, Gene expression, SNP, Platinum, HapMap

## Abstract

**Background:**

Using genome-wide genetic, gene expression, and microRNA expression (miRNA) data, we developed an integrative approach to investigate the genetic and epigenetic basis of chemotherapeutic sensitivity.

**Results:**

Through a sequential multi-stage framework, we identified genes and miRNAs whose expression correlated with platinum sensitivity, mapped these to genomic loci as quantitative trait loci (QTLs), and evaluated the associations between these QTLs and platinum sensitivity. A permutation analysis showed that top findings from our approach have a much lower false discovery rate compared to those from a traditional GWAS of drug sensitivity. Our approach identified five SNPs associated with 10 miRNAs and the expression level of 15 genes, all of which were associated with carboplatin sensitivity. Of particular interest was one SNP (rs11138019), which was associated with the expression of both miR-30d and the gene *ABCD2*, which were themselves correlated with both carboplatin and cisplatin drug-specific phenotype in the HapMap samples. Functional study found that knocking down *ABCD2 in vitro* led to increased apoptosis in ovarian cancer cell line SKOV3 after cisplatin treatment. Over-expression of miR-30d *in vitro* caused a decrease in *ABCD2* expression, suggesting a functional relationship between the two.

**Conclusions:**

We developed an integrative approach to the investigation of the genetic and epigenetic basis of human complex traits. Our approach outperformed standard GWAS and provided hints at potential biological function. The relationships between *ABCD2* and miR-30d, and *ABCD2* and platin sensitivity were experimentally validated, suggesting a functional role of *ABCD2* and miR-30d in sensitivity to platinating agents.

## Background

Platinating agents are a commonly used, highly effective family of chemotherapeutic agents. They are most frequently used to treat various solid tumors of the testes, bladder, ovaries, lung, head, and neck [1]. Response to platinating agents varies from patient to patient; furthermore, severe toxicities are common among patients receiving platinum therapy [2]. The ability to predict platinum sensitivity among patients prior to treatment has the potential to significantly improve cancer therapy. To this end, many biomarkers have been proposed to predict either response or toxicities induced by platinum [3]. However, most of these findings failed to be validated in further studies, which suggest either false discoveries or the lack of proper control for confounding (for example, the wide range of cancer types studied across varied tissue types). In this study, we developed a novel analytical framework to overcome these challenges and performed gene-knockdown and microRNA (miRNA) overexpression experiments to functionally validate our findings.

Recent advances in genomic technology have enabled genome-wide interrogation of genetic variations for their contribution to complex traits, including disease susceptibility and drug sensitivity. However, the interpretation of genome-wide association study (GWAS) findings currently remains difficult, because association tests yield little biological insight into the mechanisms for the observed relationships. To address these issues, several alternative analyses have been proposed in place of simple genotype-phenotype associations, including methods that integrate transcriptome expression quantitative trait loci (eQTL) into GWASs [4,5]. These methods not only facilitate the selection of variants with prior functional support for subsequent follow-up studies, but crucially, they propose biological mechanisms underlying the identified genotype-phenotype associations. As analytical methods that utilize high-throughput molecular phenotypes (“omics” data) to elucidate the functional basis of GWAS findings are refined, miRNAs have been increasingly recognized as an important class of regulatory molecules [6] affecting a wide variety of cellular processes [7] and playing important roles in disease pathogenesis, including cancer [8]. Consequently, we hypothesized that inclusion of this epigenetic information into large-scale association studies may yield an even finer resolution of the biological pathways underlying platinum sensitivity.

The relatively well-understood mechanism of action for platinum-based antineoplastic agents is to cause cross-linking of DNA, which results in inhibition of DNA synthesis and repair. Not surprisingly, this inhibitory activity is highly effective at disrupting proliferation of rapidly growing cell types. Although cancer cells tend to grow significantly faster than most normal tissues, variability in tumor growth as represented by disease progression is commonly observed among different types of cancer as well as among patients with the same type of cancer. Variability in cellular growth rates can be quantified, and has been shown to be a heritable trait with its own genetic and epigenetic markers [9]. Because of the platinums’ causal effect on growth, it can be difficult to tease apart the drug-specific effect from individual variability in response attributable to differential cellular growth patterns. In this study, we developed a platinum-sensitivity-specific phenotype that is independent of cellular growth rate. We hypothesized that such a phenotype would allow us to identify genetic predictors specific to platinum sensitivity.

The goal of the current study was to utilize the complex relationships between genetic variation, miRNA expression, and mRNA expression to shed light on a particular complex trait of enormous pharmacologic importance, cellular sensitivity to the platinating agents. We chose to focus on a platinum sensitivity phenotype that was measured in a set of International HapMap cell lines. These HapMap cell lines present a model system that provides publicly available genome-wide genetic as well as additional omics data, along with cytotoxicity data to derive a platinum-specific phenotype [9-13].

## Methods

### Cell lines

International HapMap YRI (Yoruba from Ibadan, Nigeria) lymphoblastoid cell lines (LCLs) were purchased from the Coriell Institute for Medical Research (Camden, NJ). Of these, 90 YRI I/II (HAPMAPPT03) samples were utilized for genome-wide discovery. LCLs were maintained in RPMI 1640/1% l-glutamine plus 15% FBS as previously described [14]. SKOV-3, an ovarian cancer cell, was purchased from ATCC and maintained according to ATCC's recommendations.

### Genotype data and expression profiling

For all cell lines, genome-wide information in the form of single nucleotide polymorphism (SNP) genotypes was downloaded from http://www.HapMap.org (ref27). Global baseline transcriptional gene expression and miRNA expression levels were quantified and reported previously [10,12] and all data were downloaded from GEO (GSE7761 and GSE34406, respectively).

### Genome-wide integrative pyramid analysis

**Figure 1:**
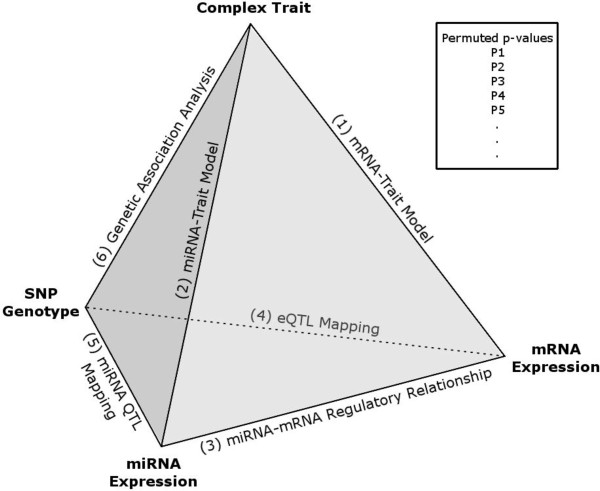
**Overview of integrative pyramid analysis schema for the genome-wide discovery.** Each stage (represented by an *edge* of the pyramid) represents a model describing the relationship between the two variables (represented by the *nodes* for the edge). Each stage uses only the data inherited from the previous stages which may have utilized certain thresholds for inclusion criteria. Thus, the filtering stages (stages 1-5) form a series of biologically relevant steps that result in a reduction in the number of SNP tested at the genetic association testing stage (stage 6) and therefore a potential increase in power. A permutation procedure (see Methods) that accounts for the fact that the samples for the filtering stages may overlap with the samples used at the genetic association testing stage was used to evaluate the overall significance of the resulting SNPs. For each replicate derived from the application of the pyramid analysis to a permuted dataset (in our analysis, n = 1000), we obtain a list of SNPs and p-values for association with the trait (designated here as *p*_*1*_, *p*_*2*_, *p*_*3*_, etc.). We utilize these sets of p-values derived from permuted datasets to estimate the null distribution.

In this study, the pyramid analysis (Figure 1) was applied to carboplatin and cisplatin separately. The pyramid analysis is a six-stage analytic framework. The first 3 stages involve identifying mRNAs and miRNAs relevant to the trait under study:

(1) $$\begin{array}{ll} Y_{i}= & \beta_{0}+\beta_{1}T_{i}+W_{i}\gamma \\ & +\epsilon_{i}\;\mathrm{with}\;\epsilon_{i}\sim N\left(0,\sigma^{2}\right)\;\left(\mathrm{mRNA}-\mathrm{trait}\;\mathrm{model}\right) \end{array}$$

(2) $$\begin{array}{ll} Y_{i}= & \lambda_{0}+\lambda_{1}M_{i}+W_{i}\gamma^{*}\\ & +{\epsilon^{*}}_{i}\;\mathrm{with}\;{\epsilon^{*}}_{i}\sim N\left(0,{\sigma^{*}}^{2}\right)\;\left(\mathrm{miRNA}-\mathrm{trait}\;\mathrm{model}\right) \end{array}$$

(3) $$\begin{array}{ll} T_{i}= & \nu_{0}+\nu_{1}M_{i}\\ & +{\epsilon^{**}}_{i}\;\mathrm{with}\;{\epsilon^{**}}_{i}\sim N\left(0,{\sigma^{**}}^{2}\right)\;\mathrm{and}\;\nu_{1}\\ & <0\;\left(\mathrm{miRNA}-\mathrm{mRNA}\;\mathrm{regulatory}\;\mathrm{relationship}\right) \end{array}$$

We identified mRNAs (e.g., in the notation above, a gene *T* such that *T*_*i*_ is the expression level for the *i*-th individual) and miRNAs (e.g., again, in the notation above, a miRNA *M* such that *M*_*i*_ is the expression level for the *i*-th individual) with expression effect (i.e., *β*_*1*_ and *λ*_*1*_ are non-zero) on the trait (stage 1 and 2). The *W*_*i*_, in this framework, provides the non-genomic covariates for the *i*-th individual. Such covariates may include ancestry (from genotype-based principal component analyses) and hidden variables (such as derived from PEER [15]. In stage 3, we further reduced the list of genes and miRNAs of interest by restricting our results to only those gene/miRNA pairs whose expression levels are negatively correlated, under the assumption that miRNA regulatory activity acts mostly through the down-regulation of gene targets. Once the list of genes and miRNAs of interest (which we refer to as “functional units”) was identified from these stages, we performed genome-wide association tests between all SNPs and the functional units assuming an additive genetic model. For the *k*-th SNP *S*_*k*_, we performed the following QTL mapping analyses:

(4) $$\begin{array}{ll} T_{i}= & \pi_{0}+\pi_{1}S_{\mathrm{ki}}+W_{i}\mu \\ & +{\epsilon^{***}}_{i}\;\mathrm{with}\;{\epsilon^{***}}_{i}\sim N\left(0,{\sigma^{***}}^{2}\right)\;\left(\mathrm{eQTL}\;\mathrm{mapping}\right) \end{array}$$

(5) $$\begin{array}{ll} M_{i}= & {\pi^{*}}_{0}+{\pi^{*}}_{1}S_{\mathrm{ki}}+W_{i}\mu^{*}\\ & +\;{\epsilon^{****}}_{i}\;\mathrm{with}\;{\epsilon^{****}}_{i}\sim N\left(0,{\sigma^{****}}^{2}\right)\;\left(\mathrm{miRNA}\;\mathrm{QTL}\;\mathrm{mapping}\right) \end{array}$$

Finally, the SNPs that were found to be associated with the functional units from these QTL mapping analyses were examined for their association with phenotype by testing H_0_: *δ*_*1*_ = 0.

(6) $$\begin{array}{ll} Y_{i}= & \delta_{0}+\delta_{1}S_{\mathrm{ki}}+W_{i}\theta \\ & +\;{\epsilon^{*****}}_{i}\;\mathrm{with}\;{\epsilon^{*****}}_{i}\sim \;N\left(0,{\sigma^{*****}}^{2}\right)\;\left(\mathrm{genetic}\;\mathrm{association}\;\mathrm{testing}\right) \end{array}$$

In our particular implementation of the pyramid analysis, we chose p < 0.05 in stages 1 and 2 to select the trait-associated genes and miRNAs. For the miRNA-mRNA negative correlations, a p-value of 10^-4^ was used, which, in our dataset, was equivalent to an FDR of 0.05 on the genome-wide scale. These filtering steps significantly decreased the number of subsequent SNP tests performed. The (potentially arbitrary) threshold chosen at each stage of the pyramid analysis may of course yield results that are not overall significant; furthermore, the filtering steps (stage 1-5) were done on the same set of samples as those on which the test (at the final stage) for SNP association was done. We therefore evaluated the overall significance of the resulting SNPs through a permutation-based false discovery rate (see next section). We should note that our method facilitates the use of a user-defined threshold at each stage of the analysis, and the permutation procedure would provide a quantification of the overall significance.

### Performance evaluation: traditional GWAS vs. pyramid analysis

To evaluate the performance of the pyramid analysis, we compared the distribution of p-values identified through traditional GWAS and that obtained from our pyramid analysis. Furthermore, to evaluate whether the inclusion of miRNA expression could yield additional performance gain, we examined the association p-value distribution for those SNPs previously associated with mRNA expression alone. Applied to the pyramid analysis, the traditional FDR approach [16], which assumes a uniform null distribution, may produce misleading results [17]. Therefore, we designed a permutation-based method that utilizes the empirical null distribution to evaluate the (empirical) FDR when multi-dimensional datasets are involved. In this method, we performed permutation analyses, permuting (n = 1000) the trait value under investigation (e.g., platinum sensitivity) while maintaining the correlation structure of the omics phenotypes and preserving the pyramid stages outlined above for each permuted dataset, to arrive at an empirical significance for the resulting list of SNPs [17]. Importantly, our permutation-based method accounts for the potentially arbitrary threshold used at each stage of the pyramid analysis.

### Platinating-agent-specific cellular sensitivity phenotypes

LCLs were treated with increasing dosage of either carboplatin or cisplatin for 72 and 48 hours, respectively. Cellular growth inhibition was measured using an Alamar Blue assay as described previously [18]. Drug concentration required to inhibit 50% of cellular growth is defined as IC_50_ and was used to characterize individual cellular sensitivity to the platinum agents. IC_50_ data was reported elsewhere [14,19] and data have been deposited into http://www.PACdb.org[20].

The intrinsic growth rate (iGrowth) of over 500 HapMap cell lines derived from a mixed-effects model has been described elsewhere [9]. We modeled a platinating agent’s IC_50_ and decomposed it as a sum of iGrowth and a drug-specific phenotype (DSP) that is independent of intrinsic growth:

$$Y_{i}=\mathrm{iGrowt}h_{i}+\mathrm{DS}P_{i}+\epsilon_{i}\;\mathrm{with}\;\epsilon_{i}\sim N\left(0,\sigma^{2}\right)$$

Thus, to obtain the DSP, a linear model was fit between iGrowth and log_2_IC_50_ with iGrowth as the independent variable. This model was fit separately for both carboplatin and cisplatin. The residuals from this model fit were used as the DSP in further analysis. Although our primary interest here was in the results of the pyramid analysis of the drug-specific component, the method can be applied to the analysis of general platinum sensitivity including cellular proliferation as well.

### Functional validation of miR-30d, ABCD2 and platinum sensitivity

We performed *ABCD2* siRNA knockdown and miR-30d over-expression experiments in SKOV-3 using DharmaFECT Transfection Reagent 1 and existing Dharmacon DharmaFECT General Transfection protocol (Thermo Scientific). siRNA for *ABCD2* (a set of 4 unique siRNAs cat. #1027416), miR-30d mimic (cat. #MSY0000245), and scramble control siRNA (AllStars Negative control, cat. #1027292) were purchased from Qiagen (Valencia, CA). Specifically, 6000 cells/well were plated in 96-well plates 24 hours prior to transfection. 100 μl of transfection media that contained 40nM siRNA, miRNA mimic, scramble control, or water (for mock transfection control), 0.4μl DharmaFECT transfection reagent 1, and standard SKOV-3 growth media were added to each well of the 96-well plate. Six hours later, transfection media was removed and replaced with regular growth media or media containing increasing concentrations of cisplatin (0uM, 5uM, and 10uM). Caspase-3 and caspase-7 activity levels were measured 24 hours post drug treatment via Caspase-Glo 3/7 Assay (Qiagen). A Student’s t-test was conducted to compare the percent caspase activity induced by cisplatin after treating the cells with either *ABCD2* siRNA, miR-30d mimic, or scramble control at each concentration. P < 0.05 was used to define statistical significance.

To confirm transfection efficiency and examine the effect of miR-30d over-expression on *ABCD2* expression, SKOV-3 cells were plated at 0.25 × 10^6^ cells/well in 6-well plates, transfected with 40nM siRNA, miRNA mimic, scramble control or water using 8 uL of DharmaFECT transfection reagent 1 in 2 mL of transfection media per well. Cells were collected in 350 uL of QIAzol Lysis Reagent (Qiagen) per well and like wells were pooled. Knockdown of *ABCD2* was confirmed by qPCR of *ABCD2* gene under the *ABCD2* siRNA, miR-30d mimic, and scramble conditions at 24 hours after transfection using Applied Biosystems Taqman primer/probe sets. miR-30d overexpression was confirmed by quantitative PCR (qPCR) using primers purchased from Exiqon (Woburn, MA). Details for total RNA isolation, cDNA conversion and PCR conditions were described previously [21]. Real-time PCR was conducted using an ABI Vii7 thermocycler (Applied Biosystems, Foster City, CA). A Student’s t-test was conducted between the scramble control treated cells and either the siRNA or mimic treated cells with p < 0.05 being considered statistically significant.

## Results

### Genome-wide integrative pyramid analysis

We developed and implemented a genome-wide analytic approach that integrates transcriptional gene expression and miRNA expression to identify robust SNP associations with IC_50_ (drug concentration required to inhibit 50% of cellular growth) in response to carboplatin or cisplatin treatment (evaluated separately). The overall workflow is illustrated in Figure 1; we refer to this throughout as a “pyramid analysis” (see Methods for detailed and precise description). The initial stage is to identify functional units that are important for the phenotype of interest (in this case, the functional units are genes and miRNAs that are negatively correlated with each other, reflecting putative regulatory relationships, and, in addition, have expression levels that are correlated with the drug sensitivity phenotype). Genetic factors that are associated with these functional units as quantitative trait loci (QTLs) [12,22,23] were identified and evaluated against the phenotype. By restricting to only those genetic variants that may be of functional significance as QTLs, we reduced the multiple testing burden in the pyramid analysis compared to traditional GWAS analysis. Furthermore, the identified genetic signals are, by definition, already related to functional units, which thus provide mechanistic hypotheses for the observed genotype-phenotype associations.

**Figure 2:**
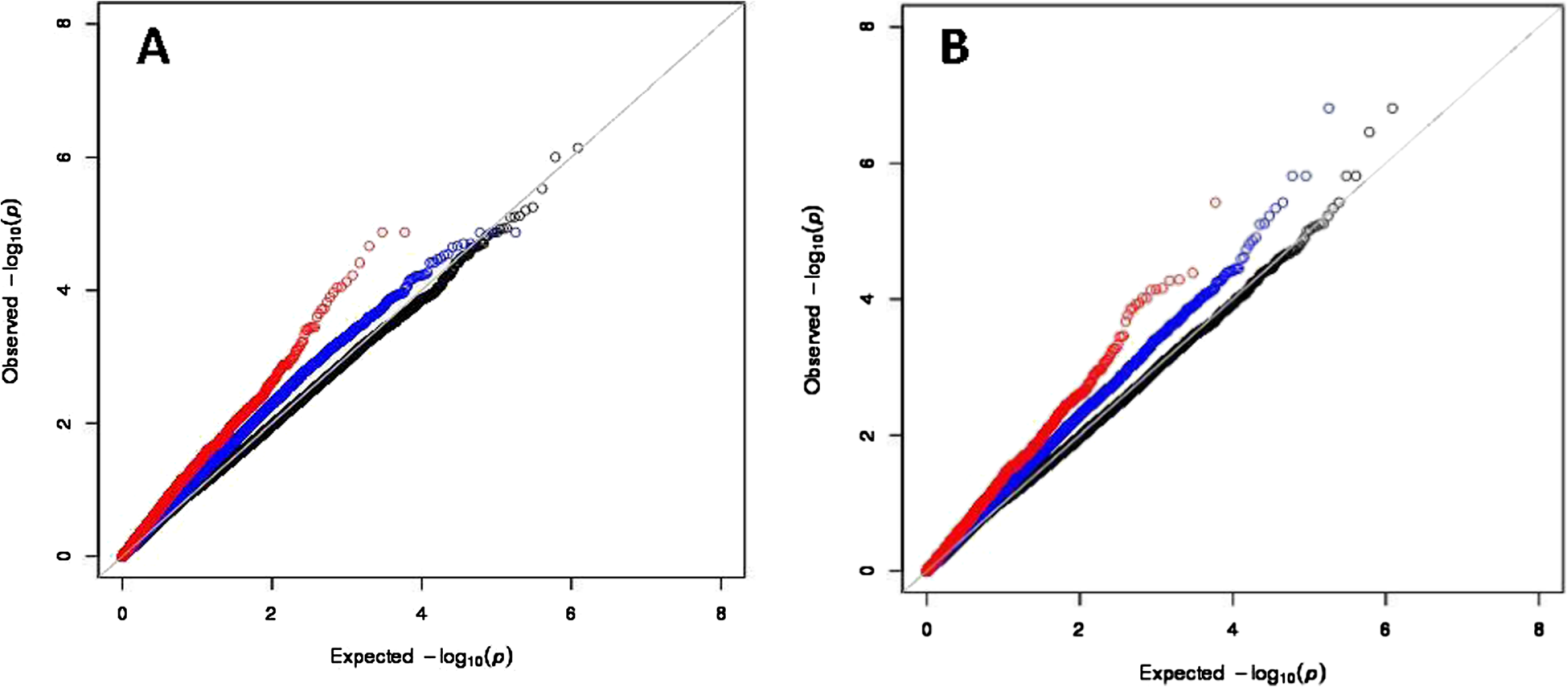
**QQ plots for association with the carboplatin and cisplatin IC**_**50**_**. A)** carboplatin; **B)** cisplatin. Black circles show the associations for all SNPs from traditional GWAS. Blue circles are the SNP associations with drug sensitivity derived from restricting the analysis to only those SNPs associated with mRNA expression. Red circles show SNP associations derived from the pyramid method.

To test the performance of this function-based approach, we compared the false discovery rate (FDR) (see Methods) for the genotype-phenotype associations derived from three different methods (namely, the traditional GWAS approach, analysis performed using only SNPs associated with gene expression as eQTLs, and the pyramid analysis as defined above) against carboplatin and cisplatin IC_50_, performed separately. As shown in Figure 2, using the SNPs filtered in stage 5 of the pyramid model (red dots), by virtue of their association with gene and miRNA expression, we identified SNPs associated with both carboplatin and cisplatin sensitivity with a lower FDR than SNPs derived from traditional GWAS or from the eQTL-based analysis. Notably, traditional GWAS yielded no significant findings due to the large number of statistical tests.

### Applying the pyramid analysis to platinum-agent-specific phenotypes

**Figure 3:**
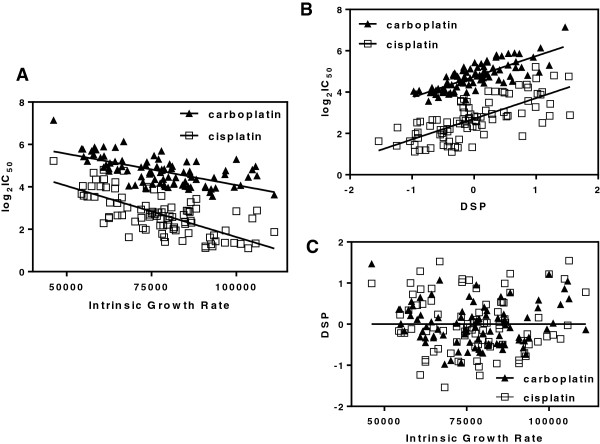
**Platinum-specific phenotype. A)** Carboplatin and cisplatin IC_50_ values vs intrinsic growth rate p <0.0001 r^2^ = 0.3912 (carboplatin) and p <0.0001 r^2^ = 0.4854 (cisplatin). **B)** Carboplatin and cisplatin drug specific phenotypes (DSPs) vs. intrinsic growth rate p = 1.0 r^2^ = 5.003 × 10^-16^ (carboplatin) and p = 1.0 r^2^ = 1.314 × 10^-18^ (cisplatin). **C)** Carboplatin and cisplatin IC_50_ values vs corresponding DSPs p <0.0001 r^2^ = 0.6088 (carboplatin) and p <0.0001 r^2^ = 0.5146 (cisplatin).

Carboplatin and cisplatin IC_50_ values were highly correlated with intrinsic growth rate (iGrowth) in the HapMap LCL samples (p <0.0001 for both carboplatin and cisplatin, Figure 3A) with faster cellular growth corresponding to lower IC_50_, representing higher sensitivity to the platinums. To control for the effect of growth rate, we developed drug-specific phenotypes (DSPs) for both carboplatin and cisplatin by using the residual of iGrowth from the IC_50_ drug sensitivity phenotype (see Methods). We observed that DSP remained highly correlated with the original platinum IC_50_ phenotype (p <0.0001, r^2^ = 0.6088 and p <0.0001, r^2^ = 0.5146 for carboplatin and cisplatin respectively, Figure 3B) but, as expected, was no longer correlated with iGrowth (p = 1.0, r^2^ = 5.003 × 10^-16^ and p = 1.0, r^2^ = 1.314 × 10^-18^ for carboplatin and cisplatin respectively, Figure 3C).

**Figure 4:**
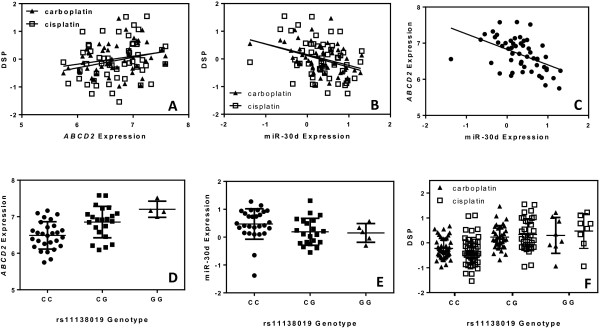
**Findings from pyramid analysis. A)***ABCD2* expression vs. carboplatin and cisplatin DSPs p = 0.019 (carboplatin) and p = 0.030 (cisplatin). **B)** miR-30d expression vs. carboplatin and cisplatin drug specific phenotypes (DSPs) p = 0.004 (carboplatin) and p = 0.020 (cisplatin). **C)** miR-30d expression vs. *ABCD2* expression p = 8.57 × 10^-5^. **D)** rs11138019 genotype vs. *ABCD2* expression p = 3.00 × 10^-5^. **E)** rs11138019 genotype vs. miR-30d expression p = 0.044. **F)** rs11138019 genotype vs. carboplatin and cisplatin DSPs p = 3.28 × 10^-5^ (carboplatin) and p = 2.60 × 10^-5^ (cisplatin).

The pyramid analysis was performed using carboplatin and cisplatin DSPs instead of IC_50_s this time. The results of these pyramid analyses are shown in Table 1. For carboplatin, we identified 5 SNPs associated with the expression of 10 miRNAs and 15 genes, each correlated with the carboplatin DSP in the discovery samples. Similarly, for cisplatin, we identified 1 SNP associated with the expression of 1 miRNA and 1 gene, each correlated with the cisplatin DSP in the discovery samples. Notably, the single SNP-miR-gene relationship (namely, rs11138019 genotype, miR-30d expression, and *ABCD2* expression) seen for cisplatin was also found for carboplatin (shown in bold in Table 2). Specifically, *ABCD2* mRNA expression was found to correlate with both DSPs in the discovery samples (Figure 4A, p = 0.019 and p = 0.030 for carboplatin and cisplatin, respectively). We also identified a positive correlation between miR-30d expression and the platinum DSPs (p = 0.004 and p = 0.020 for carboplatin and cisplatin respectively Figure 4B). *ABCD2* mRNA expression and miR-30d expression were found to be negatively correlated at p = 8.57 × 10^-5^ (Figure 4C), suggesting a regulatory relationship. The SNP rs11138019 was correlated with *ABCD2* expression at p = 3.00 × 10^-5^ (Figure 4D) and with miR-30d expression at p = 0.044 (Figure 4E). rs11138019 genotype was also correlated with both DSPs at p = 3.28 × 10^-5^ for carboplatin and p = 2.598 × 10^-5^ for cisplatin (Figure 4F).

**Table 1:** Pyramid analysis summary results for carboplatin- and cisplatin-DSP in HapMap YRI samples

	**Analysis**	**Threshold for data filtering**	**Unique number of findings**	
			**carboplatin**	**Cisplatin**
Stage 1	DSP – mRNA expression correlation	<0.05	829 genes (832 TCs)	913 genes (918 TCs)
Stage 2	DSP – miRNA expression correlation	<0.05	34 miRNA	20 miRNAs
Stage 3	Inverse correlation between miRNA and mRNA expression	<0.0001	141 genes	18 genes
			27 miRNAs	7 miRNAs
Stage 4	SNP-mRNA association	≤0.0001	51777 SNPs	9416 SNPs
			141 genes	17 genes
Stage 5	SNP-miRNA association	<0.05	38289 SNPs	5437 SNPs
			27 miRNAs	7 miRNAs
Stage 6	SNP-DSP association	≤0.0001	9 SNPs	2 SNPs
			16 genes	2 genes
			19 miRNAs	5 miRNAs
Final assembly			5 SNPs	1 SNP
			15 genes	1 gene
			10 miRNAs	1 miRNA

DSP stands for drug-specific phenotype. TC stands for transcript cluster ID.

**Table 2:** The pyramid analysis results for carboplatin in HapMap YRI samples

**SNP**	**miRNA**	**mRNA**	**SNP-phenotype pvalue**	**SNP-miRNA pvalue**	**SNP-mRNA pvalue**	**miRNA-phenotype pvalue**	**miRNA-mRNA pvalue**	**mRNA-phenotype pvalue**
**rs11138019**	hsa-miR-181a	*ABCD2*	3.28E-05	7.22E-03	3.00E-05	6.93E-03	3.47E-06	1.88E-02
		*DDR2*			1.00E-05		4.00E-02	5.72E-03
		*ECOP*			1.00E-04		5.91E-03	2.31E-03
		*EIF2AK3*			1.00E-05		2.00E-02	1.54E-02
		*HMOX1*			1.00E-04		1.65E-04	3.76E-02
		*HSPC159*			7.00E-05		1.10E-04	1.27E-02
		*LARGE*			5.00E-05		1.96E-05	1.05E-03
		*PDK2*			3.00E-05		1.49E-04	2.44E-02
		*PIK3R5*			1.00E-04		4.32E-03	1.86E-02
		*RBM47*			1.00E-04		1.00E-02	5.55E-03
		*USP53*			2.00E-05		8.56E-05	4.77E-02
	hsa-miR-181b	*ABCD2*		1.26E-02	3.00E-05	1.17E-02	4.70E-06	1.88E-02
		*ECOP*			1.00E-04		3.38E-03	2.31E-03
		*EIF2AK3*			1.00E-05		2.00E-02	1.54E-02
		*HMOX1*			1.00E-04		3.56E-04	3.76E-02
		*HSPC159*			7.00E-05		5.84E-04	1.27E-02
		*LARGE*			5.00E-05		6.74E-05	1.05E-03
		*MEF2D*			4.00E-05		4.00E-02	7.53E-03
		*PDK2*			3.00E-05		1.81E-04	2.44E-02
		*PIK3R5*			1.00E-04		4.60E-03	1.86E-02
		*RBM47*			1.00E-04		1.00E-02	5.55E-03
		*USP53*			2.00E-05		8.32E-05	4.77E-02
	hsa-miR-19b	*ECOP*		2.71E-02	1.00E-04	9.82E-04	1.00E-02	2.31E-03
		*EIF2AK3*			1.00E-05		1.76E-03	1.54E-02
		*HMOX1*			1.00E-04		1.46E-03	3.76E-02
		*LARGE*			5.00E-05		4.00E-02	1.05E-03
		*MEF2D*			4.00E-05		2.35E-03	7.53E-03
		*PDK2*			3.00E-05		1.00E-02	2.44E-02
		*RBM47*			1.00E-04		5.92E-03	5.55E-03
	hsa-miR-20b	*ABCD2*		2.90E-02	3.00E-05	9.45E-05	1.00E-02	1.88E-02
		*DDR2*			1.00E-05		2.00E-02	5.72E-03
		*ECOP*			1.00E-04		4.76E-04	2.31E-03
		*EIF2AK3*			1.00E-05		3.41E-05	1.54E-02
		*HMOX1*			1.00E-04		2.55E-03	3.76E-02
		*HSPC159*			7.00E-05		2.00E-02	1.27E-02
		*LARGE*			5.00E-05		1.93E-04	1.05E-03
		*MEF2D*			4.00E-05		1.11E-04	7.53E-03
		*PDK2*			3.00E-05		2.38E-05	2.44E-02
		*PIK3R5*			1.00E-04		8.33E-03	1.86E-02
		*RBM47*			1.00E-04		4.48E-04	5.55E-03
		*USP53*			2.00E-05		3.00E-02	4.77E-02
	**hsa-miR-30d**	***ABCD2***		4.42E-02	3.00E-05	3.80E-03	8.57E-05	1.88E-02
		*DDR2*			1.00E-05		3.55E-05	5.72E-03
		*ECOP*			1.00E-04		3.52E-03	2.31E-03
		*HMOX1*			1.00E-04		3.09E-03	3.76E-02
		*HSPC159*			7.00E-05		4.68E-04	1.27E-02
		*LARGE*			5.00E-05		1.00E-02	1.05E-03
		*PDK2*			3.00E-05		1.00E-02	2.44E-02
		*PIK3R5*			1.00E-04		1.46E-03	1.86E-02
		*USP53*			2.00E-05		3.12E-04	4.77E-02
	hsa-miR-363	*ABCD2*		1.28E-02	3.00E-05	1.13E-03	1.74E-04	1.88E-02
		*DDR2*			1.00E-05		3.94E-05	5.72E-03
		*ECOP*			1.00E-04		3.00E-02	2.31E-03
		*EIF2AK3*			1.00E-05		4.00E-03	1.54E-02
		*HSPC159*			7.00E-05		1.35E-03	1.27E-02
		*LARGE*			5.00E-05		1.07E-05	1.05E-03
		*MEF2D*			4.00E-05		1.00E-02	7.53E-03
		*PDK2*			3.00E-05		1.36E-04	2.44E-02
		*PIK3R5*			1.00E-04		1.66E-06	1.86E-02
		*USP53*			2.00E-05		3.99E-06	4.77E-02
rs4919716	hsa-miR-20b	*ECOP*	1.95E-05	3.00E-02	9.00E-05	9.45E-05	4.76E-04	2.31E-03
		*FURIN*			1.00E-04		3.00E-02	4.27E-02
		*MTMR14*			8.00E-05		7.63E-03	1.73E-02
	hsa-miR-30b	*ECOP*		4.09E-02	9.00E-05	7.08E-03	3.57E-03	2.31E-03
		*FURIN*			1.00E-04		2.43E-05	4.27E-02
		*MTMR14*			8.00E-05		5.77E-03	1.73E-02
	hsa-miR-30d	*ECOP*		2.10E-02	9.00E-05	3.80E-03	3.52E-03	2.31E-03
		*FURIN*			1.00E-04		1.69E-05	4.27E-02
		*MTMR14*			8.00E-05		1.00E-02	1.73E-02
	hsa-miR-339-5p	*ECOP*		1.12E-02	9.00E-05	1.31E-02	9.37E-03	2.31E-03
		*FURIN*			1.00E-04		2.36E-03	4.27E-02
		*MTMR14*			8.00E-05		3.42E-03	1.73E-02
	hsa-miR-363	*ECOP*		2.64E-02	9.00E-05	1.13E-03	3.00E-02	2.31E-03
		*FURIN*			1.00E-04		2.33E-06	4.27E-02
		*MTMR14*			8.00E-05		5.69E-04	1.73E-02
rs7670734	hsa-let-7i	*TP63*	5.21E-05	2.20E-02	8.00E-05	2.44E-03	5.08E-05	3.95E-02
	hsa-miR-20b	*TP63*		4.26E-02		9.45E-05	1.00E-02	
	hsa-miR-25	*TP63*		1.11E-02		1.65E-02	1.00E-02	
	hsa-miR-339-5p	*TP63*		1.07E-02		1.31E-02	5.44E-04	
	hsa-miR-363	*TP63*		2.48E-02		1.13E-03	1.76E-06	
rs7677704	hsa-let-7i	*TP63*	5.21E-05	2.20E-02	8.00E-05	2.44E-03	5.08E-05	3.95E-02
	hsa-miR-20b	*TP63*		4.26E-02		9.45E-05	1.00E-02	
	hsa-miR-25	*TP63*		1.11E-02		1.65E-02	1.00E-02	
	hsa-miR-339-5p	*TP63*		1.07E-02		1.31E-02	5.44E-04	
	hsa-miR-363	*TP63*		2.48E-02		1.13E-03	1.76E-06	
rs9312445	hsa-let-7i	*TP63*	5.21E-05	2.20E-02	8.00E-05	2.44E-03	5.08E-05	3.95E-02
	hsa-miR-20b	*TP63*		4.26E-02		9.45E-05	1.00E-02	
	hsa-miR-25	*TP63*		1.11E-02		1.65E-02	1.00E-02	
	hsa-miR-339-5p	*TP63*		1.07E-02		1.31E-02	5.44E-04	
	hsa-miR-363	*TP63*		2.48E-02		1.13E-03	1.76E-06	

Overall significance was evaluated using permutations.

*Overlap with cisplatin pyramid analysis is shown in bold.

### Functional validation of miR-30d, ABCD2 and platinum sensitivity

**Figure 5:**
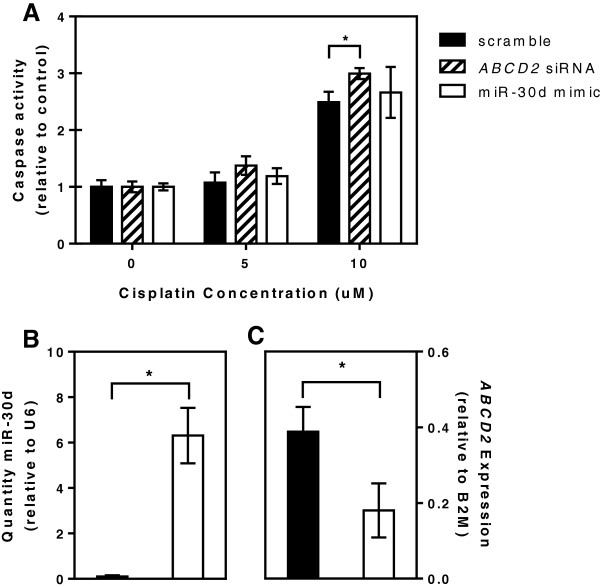
**Functional validation of *ABCD2 *and miR-30d. A)** Increase in cisplatin-induced apoptosis 24 hours after transfection with 40nM *ABCD2* siRNA as compared to scrambled control siRNA (p = 0.013). **B)** Increased miR-30d expression 24 hours after transfection with 40nM miR-30d mimic (normalized to RNU6 housekeeping control) compared to scrambled control siRNA (p = 0.001). **C)** Decreased *ABCD2* expression 24 hours after transfection with miR-30d mimic (normalized to *B2M* housekeeping control) compared to scramble control siRNA (p = 0.047); data not shown for *ABCD2* siRNA as remaining amount of *ABCD2* mRNA was no longer quantifiable by qPCR 24 hours after transfection.

We conducted a gene knockdown experiment of *ABCD2* in SKOV-3 in which the addition of *ABCD2* siRNA resulted in decreased expression of *ABCD2* mRNA compared to control (data not shown; after transfection with 40nM *ABCD2* siRNA, *ABCD2* expression was no longer quantifiable by qPCR) and consequently increased cisplatin-induced cellular apoptosis, as shown by increased caspase3/7 activity, after treatment with 10 μM cisplatin (Figure 5A, p = 0.013). When SKOV-3 cells were transfected with miR-30d mimic, we observed an increase in miR-30d expression (Figure 5B, p = 0.001) and a decrease in *ABCD2* expression (Figure 5C, p = 0.047). Cisplatin-induced cellular apoptosis showed a trend in the same direction as with *ABCD2* siRNA, however the effect was not significant (p > 0.05).

## Discussion

Building on the successes of GWAS for mapping complex traits, considerable efforts have been invested in the development of new methods to decipher the contribution of genetic variants that have modest effects on phenotype. One reasonable approach is to prioritize SNPs with prior functional support from increasingly available omics studies. This method is supported by previous studies that demonstrated that reproducible GWAS findings for human complex traits are more likely to fall in loci with gene/miRNA regulatory effects in the form of mRNA and miRNA expression quantitative trait loci (m-eQTLs and mi-eQTLs) [4,5,12]. In this study, utilizing multiple high-throughput genome-wide datasets, we developed an integrative approach that incorporates mRNA and miRNA regulatory elements as well as genetic variation to gain mechanistic insight into platinum sensitivity.

To compare the performance of our integrative pyramid analysis to that of traditional GWAS, we compared the p-value distribution between these analytic approaches. We found that the use of our pyramid approach, which prioritizes SNPs with prior functional support from studies of high-throughput molecular phenotypes (i.e., both mRNA and miRNA expression), substantially improved power to detect significant associations with platinum sensitivity. Importantly, for both carboplatin and cisplatin, the use of mRNA data in defining functionally important SNPs for inclusion in downstream analyses performed better than the simple use of whole-genome SNP data, and the further refinement with the inclusion of miRNA expression information further improved performance. Indeed, at FDR < 0.10, no significant SNP association with cisplatin IC_50_ was found under traditional GWAS; however, using the pyramid analysis, we identified 2 SNPs significantly associated with cisplatin sensitivity at FDR < 0.10, and, indeed, the most significant SNP reached FDR < 0.05. These results demonstrate the substantial gain in power from an application of the pyramid analysis, at least for some traits, despite the small sample size. We realize that some variants may be missed by the method proposed here, which explicitly enriches for SNPs regulating miRNAs and mRNAs with the additional requirement that the miRNA and mRNA are in a specific regulatory relationship. Nevertheless, the substantially improved signals from our analyses relative to traditional unbiased GWAS, for both platinating agents, suggest the importance of these regulatory relationships underlying platinum sensitivity.

Over the years, numerous studies have been conducted to discover robust predictors of platinum response and/or toxicity. Unfortunately, many of the identified biomarkers have failed to replicate under further interrogation. One of the reasons that this may be the case is that, due to its presumed mechanism of action, the efficacy of platinating agents is highly associated with patterns of cellular growth, which may lead to very variable results across multiple cell and tissue types. Since the platinums are used in the treatment of a wide variety of cancer types, an approach that separates platinum-specific effects and the intrinsic growth rates of different tumors may provide a more robust phenotype in identifying biomarkers that can predict platinum sensitivity independent from cell types. In this study, we developed a DSP which accounted for the contribution of growth rate in cellular response to cisplatin and carboplatin in our discovery cell lines. Given the enormous amount of genomic information available, utilizing the HapMap LCLs as our discovery set allowed us to generate this new and novel phenotype, which would be difficult in a smaller or less homogenous sample set.

Through our integrative pyramid analysis, we identified a SNP (rs11138019) associated with the expression of miR-30d and *ABCD2*; importantly, the SNP, miRNA and mRNA correlated with both carboplatin- and cisplatin- specific sensitivity in the HapMap LCL samples. miR-30d has been shown to play an important role in several biological processes related to cancer development and progression. For example, miR-30d has been suggested to be an oncomir, regulating tumor cell proliferation [24], and senescence through regulation of tumor suppressor gene *p53*[25]. miR-30d is associated with poor clinical outcomes in ovarian cancer patients [26], and has been identified as a potential prognostic marker of prostate cancer [27]*.* It has also been shown to enhance invasion and immuno-suppression during metastasis through regulation of GalNAx transferases [28]. A recent study reported that miR-30d was amplified in more than 30% of multiple types of human solid tumors [26], suggesting a therapeutic role of the platinums in these cancers.

In our study, we did not observe significantly increased platinum-induced apoptotic activity 24 hours after over-expressing miR-30d in SKOV-3 cells; however we did observe decreased *ABCD2* expression 24 hours after miR-30d over-expression. The decrease in *ABCD2* following miR-30d over-expression was of much smaller magnitude than the decrease in *ABCD2* after using targeted siRNA. It is likely that the moderate effect of miR-30d on *ABCD2* expression was not enough to induce a phenotypic change large enough to be measurable with our endpoint assay, whereas the sensitizing effect could be seen when directly manipulating expression levels of *ABCD2* through siRNA. Measurement of apoptotic activity was chosen as the cellular phenotype in our functional studies because, unlike common cellular proliferation assays (AlamarBlue and Celltiter Glo), it is a measurement of drug-specific effect, unaffected by cellular growth rate.

Our study also identified a potential gene target of miR-30d, *ABCD2* (ATP-Binding Cassette Sub-Family D Member 2). *ABCD2* is a member of the superfamily of ATP-binding cassette transporters (which transport various molecules across extra- and intra-cellular membranes) and of the ALD subfamily (involved in peroxisomal import of fatty acids and/or fatty acyl-CoAs in the organelle). While its function is unknown, *ABCD2* is the closest homologue of *ABCD1,* the mutation of which is the well known cause of Zellweger Syndrome [29]. However, mutation in *ABCD2* has been shown not to confer the same loss-of-function-related disease state [29,30]. *ABCD2* has also been shown to interact directly with PEX19 (peroxisomal biogenesis factor 19) [31], β-catenin, and TCF-4 [32]. To gain insight into the function of *ABCD2* in cancer, we performed gene knockdown experiments in an ovarian cancer cell line, SKOV-3. We found that reduction of *ABCD2* expression by siRNA knockdown led to an increase in apoptotic activity after treatment with cisplatin, suggesting that *ABCD2* plays a role in cellular sensitivity to the platinums in ovarian cancers. These results are consistent with a recent study which has shown that *ABCD2* is part of a gene expression signature with statistically significant correlation with overall survival in ovarian carcinoma patients with effusions [33]. Additional studies on the clinical relevance of our findings are therefore warranted.

## Conclusions

In summary, we developed an integrative model that incorporates multi-dimensional genomics datasets (genotype, mRNA, miRNA) to identify genetic variants associated with platinum sensitivity through their regulatory effect on the transcriptome. This novel model can be applied to the study of other complex traits. Through our pyramid analysis, we discovered a set of SNPs, miRNAs, and genes which potentially comprise a functionally important regulatory pathway in cellular sensitivity to the platinating agents. Functional testing supports the function of *ABCD2* and potentially miR-30d in conferring platinum resistance.

## Competing interests

The authors declare that they have no competing interests.

## Authors’ contributions

BL carried out the functional studies, participated in data analysis, and drafted the manuscript. ERG conducted the bioinformatics research, carried out the statistical analyses, and participated in drafting the manuscript. DL participated in the functional studies and data analysis. HI participated in the statistical analyses and development of the growth phenotype. PG designed the drug specific phenotype (DSP). DZ participated in gene expression studies. NC helped in study design. RSH conceived of the study, participated in its design, coordination, funding, and manuscript preparation. All authors read and approved the final manuscript.
